# Supplementary material for: The efficacy of the fixed combination of latanoprost and timolol versus other fixed combinations for primary open-angle glaucoma and ocular hypertension: A systematic review and meta-analysis
Source: PLoS One. 2020 Feb 27;15(2):e0229682. doi: 10.1371/journal.pone.0229682 (PMC7046276; doi:10.1371/journal.pone.0229682)
Supplement: S2 File — (DOC) [file pone.0229682.s002.doc]

1. MEDLINE ( PubMed) search strategy

#51 Search ((#11) AND #20) AND #49

#50 Search (#47) OR #32

#49 Search (#47) OR #48

#48 Search ((#21) OR #22) AND ((#23) OR #25)

#47 Search ((((((((((( #34) OR #35) OR #36) OR #37) OR #38) OR #39) OR #40) OR #41) OR #42) OR #43) OR #44) OR #46

#46 Search fixed-combination latanoprost/timolol

#44 Search fixed latanoprost/timolol combined therapy

#43 Search latanoprost 0:005%/timolol 0.5%

#42 Search latanoprost/timolol maleate combination

#41 Search fixed combinations of latanoprost plus timolol

#40 Search fixed-combination of latanoprost 0.005% and timolol 0.5%

#39 Search latanoprost-timolol fixed combination

#38 Search latanoprost/timolol fixed combination

#37 Search Fixed combination of latanoprost and timolol

#36 Search latanoprost/timolol, fixed combinations

#35 Search fixed combinations of latanoprost/timolol

#34 Search the fixed combination of latanoprost/timolol

#32 Search (#31) AND #30

#31 Search (((#23) OR #25) AND #22) AND #21

#30 Search (((#26) OR #27) OR #28) OR #29

#29 Search (Timolol[MeSH Terms]) AND adverse effects[MeSH Subheading]

#28 Search (Timolol[MeSH Terms]) AND therapeutic use[MeSH Subheading]

#27 Search ((Prostaglandins F, Synthetic[MeSH Terms]) AND adverse effects[MeSH Subheading]

#26 Search (((Prostaglandins F, Synthetic[MeSH Terms]) AND therapeutic use[MeSH Subheading]) #25 Search drug combinations[MeSH Terms]

#23 Search Drug Therapy, Combination[MeSH Terms]

#22 Search Antihypertensive Agents[MeSH Terms]

#21 Search Ophthalmic Solutions[MeSH Terms]

#20 Search (((((#13) OR #15) OR #16) OR #17) OR #18) OR #19

#19 Search ocular hypertensive[Title/Abstract]

#18 Search ocular hypertension[Title/Abstract]

#17 Search primary open-angle glaucoma[Title/Abstract]

#16 Search open-angle glaucoma[Title/Abstract]

#15 Search "Ocular Hypertension"[Mesh]

#13 Search "Glaucoma, Open-Angle"[Mesh]

#11 Search #9 not #10

#10 Search animals[MeSH Terms] NOT ((animals[MeSH Terms]) AND humans[MeSH Terms])

#9 Search #1 or #2 or #3 or #4 or #5 or #6 or #7 or #8

#8 Search groups[Title/Abstract]

#7 Search trial[Title/Abstract]

#6 Search randomly[Title/Abstract]

#5 Search drug therapy[MeSH Subheading]

#4 Search placebo[Title/Abstract]

#3 Search randomized[Title/Abstract]

#2 Search controlled clinical trial[Publication Type]

#1 randomized controlled trial[Publication Type])

2 EMBASE (Ovid) search strategy

#42. #17 AND #21 AND #41

#41. #32 OR #33 OR #34 OR #35 OR #36 OR #40

#40. #25 AND #39

#39. #27 OR #29

#38. #17 AND #21 AND #37

#37. #31 OR #32 OR #33 OR #34 OR #35 OR #36

#36. 'fixed combinations of latanoprost plus timolol'

#35. 'fixed-combination of latanoprost 0.005% and timolol 0.5%'

#34. 'the fixed combination of latanoprost/timolol'

#33. 'fixed-combination latanoprost/timolol'

#32. 'latanoprost plus timolol'/exp

#31. #25 AND #30

#30. #27 AND #29

#29. #26 OR #28

#28. 'latanoprost'/exp

#27. 'timolol'/exp

#26. 'prostaglandin f'/exp

#25. #22 OR #23 OR #24

#24. 'antihypertensive agent'/exp

#23. 'eye drops'/exp

#22. 'drug combination'/exp

#21. #18 OR #19 OR #20

#20. 'ocular hypertensive'

#19. 'intraocular hypertension'/exp

#18. 'open angle glaucoma'/exp

#17. #1 OR #2 OR #3 OR #4 OR #5 OR #6 OR #7 OR #8 OR#9 OR #10 OR #11 OR #12 OR #13 OR #14 OR #15 OR#16

#16. 'crossover procedure'/exp

#15. 'single blind procedure'/exp

#14. 'double blind procedure'/exp

#13. volunteer*

#12. assign*

#11. singl*

#10. blind*

#9. placebo*

#8. crossover*

#7. factorial*

#6. random*

#5. doubl*

#4. doubl* AND blind*

#3. blind* AND singl*

#2. allocat*

#1. 'randomized controlled trial'/exp

3 CENTRAL search strategy

#1(Glaucoma, Open-Angle):kw or (Glaucoma, Open-Angle):ti,ab,kw and (Ocular Hypertension):kw and (Ocular Hypertension):ti,ab,kw

#2MeSH descriptor Ocular Hypertension, this term only

#3MeSH descriptor Glaucoma, Open-Angle, this term only

#4(#1 OR #2 OR #3

)#5(Drug Combinations):kw or (Ophthalmic Solutions):kw or (Antihypertensive Agents):kw

#6(Prostaglandins F, Synthetic):kw and (Timolol):kw

#7MeSH descriptor Timolol, this term only

#8MeSH descriptor Prostaglandins F, Synthetic, this term only

#9(Fixed combination of latanoprost and timolol):ti,ab,kw or (latanoprost/timolol maleate combination):ti,ab,kw or fixed combinations of latanoprost plus timolol:ti,ab,kw or (fixed latanoprost/timolol combined therapy):ti,ab,kw or (fixed-combination of latanoprost 0.005% and timolol 0.5%）:ti,ab,kw

#10(#6 OR #7 OR #8 OR #9)

#11(#10 AND #5)

#12(#4 AND #11)

4 WOS search strategy

#1 TS=(glaucoma, open-angle) OR TI=(open angle galucoma)

# 2 topic=(ocular hypertension) OR title=(ocular hypertension)

# 3 topic=(drug combinations) OR topic=(ophthalmic solutions) OR topic=(antihypretensive agents)

# 4 topic=(Prostaglandins F, Synthetic) OR topic=(timolol

# 5 #2 OR #1

# 6 #4 AND #3

# 7 title=(Fixed combination of latanoprost and timolol) OR title=(latanoprost/timolol maleate combination) OR title=(fixed combinations of latanoprost plus timolol) OR title=(fixed latanoprost/timolol combined therapy) OR title=(fixed-combination of latanoprost 0.005% and timolol 0.5%)

# 8 topic=(the fixed combination of latanoprost and timolol)

# 9 #6 OR #7 OR #8

# 10 TS=(human) NOT TS=(animal)

# 11 TS=(trial or random* or experiment*) OR TS=(evaluat*) OR TS=(random* control* trial)

# 12 #11 AND #10

# 13 #12 AND #9 AND #5

# 14 TS=(human) NOT TS=(animal)

# 15 TS=(trial or random* or experiment*) OR TS=(evaluat*) OR TS=(random* control* trial)

# 16 #15 AND #14

# 17 #16 AND #12 AND #7

# 18 #16 AND #13 AND #7
