# Supplementary material for: The efficacy of the fixed combination of latanoprost and timolol versus other fixed combinations for primary open-angle glaucoma and ocular hypertension: A systematic review and meta-analysis
Source: PLoS One. 2020 Feb 27;15(2):e0229682. doi: 10.1371/journal.pone.0229682 (PMC7046276; doi:10.1371/journal.pone.0229682)
Supplement: Attachment — Submitted filename: Response to Reviewers.docx [file pone.0229682.s003.docx]

Dear Editor:

Thank you very much for your letter and the reviewers for their comments concerning our manuscript entitled “The efficacy of the fixed combination of latanoprost and timolol versus other fixed combinations for primary open-angle glaucoma and ocular hypertension: a systematic review and meta-analysis”(PONE-D-19-26644). The comments from the reviewers are the most helpful in revision of our manuscript. We have studied comments carefully, and made revision according to the reviewers’ suggestion. Revised portion are marked in red in the revised manuscript. The responses to the reviewers’ comments are as following:

**Reviewer #1:**

*This is a well-done study in which the authors obtained interesting results, even if some of them were already known. This metaanalysis outlined the efficacy of fixed combination.*

*There are some English tense to correct, but the manuscript is well written.*

**Response:** We have checked and corrected English tense errors in the manuscript.

**Reviewer #2:**

1. *Line 83. There are more fixed combination (brinzolamide/timolol, brimonidine/brinzolamide and the oldest pilocarpine/timolol). Why did you forget all these?*

**Response:** Special thanks for your good comments. We have added these fixed combinations in the introduction. We have reported that only four types of fixed combinations were found in the control groups of the included studies in the systematic review.

2. *Line 384. Please change DTFC according with other abbreviations. Really, I would prefer all the abbreviations as DTFC than FCDT…*

**Response:** We have accepted your suggestion and have modified the abbreviations of all fixed combinations.

3. *Line 442. Please avoid “To the best of our knowledge” and “first”. You can’t read all the published Literature and all the databases.*

**Response:** We have modified this section according to the recommendations.

We hope that the revised manuscript is now acceptable for publication in your journal.

With kind regards.

Shaohua Huang

medhsh@163.com
